# Automatic electrode scalar location assessment after cochlear implantation using a novel imaging software

**DOI:** 10.1038/s41598-023-39275-3

**Published:** 2023-07-31

**Authors:** S. Geiger, M. Iso-Mustajärvi, T. Nauwelaers, E. Avci, P. Julkunen, P. Linder, T. Silvast, A. Dietz

**Affiliations:** 1 Advanced Bionics, European Research Center, Hannover, Germany; 2 Department of Otorhinolaryngology, Kuopio University Hospital, Kuopio, Finland; 3 Department of Technical Physics, University of Eastern Finland, Kuopio, Finland; 4 Department of Clinical Neurophysiology, Kuopio University Hospital, Kuopio, Finland; 5 SIB Labs, Dempartment of Technical Physics, University of Eastern Finland, Kuopio, Finland

**Keywords:** Medical imaging, Software

## Abstract

As of today, image-based assessment of cochlear implant electrode array location is not part of the clinical routine. Low resolution and contrast of computer tomography (CT) imaging, as well as electrode array artefacts, prevent visibility of intracochlear structures and result in low accuracy in determining location of the electrode array. Further, trauma assessment based on clinical-CT images requires a uniform image-based trauma scaling. Goal of this study was to evaluate the accuracy of a novel imaging software to detect electrode scalar location. Six cadaveric temporal bones were implanted with Advanced Bionics SlimJ and Mid-Scala electrode arrays. Clinical-CT scans were taken pre- and postoperatively. In addition, micro-CTs were taken post-operatively for validation. The electrode scalar location rating done by the software was compared to the rating of two experienced otosurgeons and the micro-CT images. A 3-step electrode scalar location grading scale (0 = electrode in scala tympani, 1 = interaction of electrode with basilar membrane/osseous spiral lamina, 2 = translocation of electrode into scala vestibuli) was introduced for the assessment. The software showed a high sensitivity of 100% and a specificity of 98.7% for rating the electrode location. The correlation between rating methods was strong (kappa > 0.890). The software gives a fast and reliable method of evaluating electrode scalar location for cone beam CT scans. The introduced electrode location grading scale was adapted for assessing clinical CT images.

## Introduction

The cochlear implant (CI) has become a standard treatment option for individuals with severe or profound hearing loss. Recent development in surgical technique and electrode design have been primarily aimed at preserving the delicate inner ear structures, which may even conserve the CI recipient’s residual hearing^1–3^. Even without residual hearing, electrode dislocation from the scala tympani (ST) to the scala media (SM) and scala vestibuli (SV) has been shown to negatively influence postoperative hearing outcomes with the CI^4–6^. Histopathologic studies have shown that translocation is more likely to cause fibrosis and neural degeneration in the cochlea as compared to full ST insertions without translocation^7,8^. Therefore, a reliable postoperative trauma assessment of the cochlea is a crucial component for both preclinical and clinical CI studies that aim to characterize electrode array behaviour.

In preclinical studies, verification of electrode location and trauma analysis can be performed using a variety of techniques. Classically, histological analysis of the cochlea has been the “gold standard” for evaluating the results of electrode insertions in cadaveric temporal bones (TB). For histological studies, the trauma grading scale established by Eshragi et al.^9^ has been commonly used and classifies trauma into the following four categories: 0 = no trauma, 1 = lifting of the basilar membrane (BM), 2 = rupture of the BM, 3 = dislocation of the electrode and 4 = fracture of the osseus spiral lamina. With the development of new imaging modalities, especially cone-beam computed tomography (CBCT), CT is increasingly being used in TB studies^10,11^. Furthermore, with high resolution micro-computed tomography (Micro-CT), internal cochlear structures become visible and the electrode location as well as trauma assessment can be done more precisely^12,13^.

For the clinical setting, X-ray, multiplanar CT, and CBCT are currently the modalities used for postoperative imaging. In addition to a manual assessment by experienced observers^4,14,15^, software-based evaluations such as fusion imaging techniques^16–18^ and automatic or semi-automatic cochlea and electrode model generation^19–26^ are used to obtain a more accurate assessment of the electrode array’s location. Since the BM is not often visible on CBCTs it is reconstructed based on the size and shape of cochlea^20^, on the average location of the BM^17^, on atlas-based approaches^23,24,26^ or statistical-deformation-modelling^25^. In the study of Sipari et al.^27^, a modified Eshragi grading was used with fusion imaging (i.e., combining preoperative and postoperative images) and compared to histology, returning a sensitivity of 87.5% and a specificity of 97.3%. With the various clinically applicable options currently available for CI trauma assessment, a uniform image-based trauma scaling system is needed for clinics as well as for future CI research.

Within this study, we evaluated the accuracy of a novel imaging software which allows detection of the electrode array’s scalar location through the analysis of clinical CBCT images. In order to quantify this analysis, a novel scalar location scale has been introduced. The results of the software analysis were compared with experts’ assessments and micro-CT images.

## Methods

### Temporal bones and surgery

Six cadaveric human temporal bones were used for this study. The temporal bones were collected within 24 h of death and immediately frozen. The study fulfilled the Helsinki Declaration for Ethical use of human materials. The study was conducted according to Finnish legislation and institutional approval was granted by the Kuopio University Hospital (No. 5551883), and the Finnish Ministry of Social Affairs and Health authorized the use of cadaveric TBs (No. 9202/06.01.03.01/2013). The anonymity of participants was guaranteed and informed consent was obtained from all participants. Before electrode array insertion, the temporal bones were thawed and brought to room temperature. A partial mastoidectomy with posterior tympanotomy was then performed. All insertions were made via the round window membrane (RW), with the aim of complete insertion. In case of resistance, the insertion angle was altered. If resistance still occurred on the second attempt, the insertion depth was limited to this point. All temporal bones were implanted with the HiFocus SlimJ (SlimJ) or HiFocus MidScala (MS) electrode arrays from Advanced Bionics LLC (Valencia, USA). These electrode arrays consist of 16 active contacts and one inactive marker contact. The HiFocus SlimJ electrode array is a lateral wall design, the HiFocus MidScala is an electrode array designed to sit in the middle of the scala tympani duct. Both electrode arrays target an insertion angle of 420 degrees^28^ in an average-sized cochlea. Table 1 shows a summary of the six temporal bones with the side, electrode array implanted and the main dimensions, *A* and *B* as described by Escude et al.^29^. *A* is defined as the length from the round window through the modiolar axis to the lateral wall. *B* is the length measured perpendicular to *A* through the modiolar axis and defines the distance between the lateral walls.

**Table 1:** This table shows the six temporal bones used for the study.

Temporal bone	Side	Electrode	A [mm]	B [mm]
TB1	Right	MS	9.1	6.6
TB2	Right	SlimJ	8.4	6.7
TB3	Right	MS	9.5	7.1
TB4	Right	MS	9.0	6.9
TB5	Right	SlimJ	9.2	7.0
TB6	Right	SlimJ	9.2	7.4

All temporal bones were right cochleae. Half of the implanted electrode arrays were Advanced Bionics HiFocus SlimJ (SlimJ) electrodes and the other half Advanced Bionics HiFocus MidScala (MS) electrodes. Cochlea A and B dimensions are given for each temporal bone.

#### CT and microCT scanning technique

A clinical CBCT (ProMax 3D Max, Planmeca Oy, Helsinki, Finland) was performed for each bone, both preoperatively and following insertion of the electrode array. The scanning protocol for the preoperative CBCT was as follows: tube voltage 80 kV, tube current 16 mA, imaging time 15 s and FOV 50 × 55 mm. For the postoperative CBCT scan the protocol was as follows: tube voltage 96 kV, tube current 7 mA, imaging time 15 s, and FOV 50 × 55 mm. The axial, sagittal and coronal slices of the CBCTs were reconstructed using Planmeca Romexis software, with a 100 µm isometric voxel size.

In addition, a postoperative high-resolution micro-CT (XT H 225, Nikon Metrology NV, Leuven, Belgium) scan was performed to identify the exact location of the electrode array in relation to the intracochlear structures. Prior to the micro-CT scan, the perilymph was carefully aspirated under a surgical microscope to enhance image contrast. The scanning protocol for the postoperative micro-CT scan was the following: tube voltage 60 kV, tube current 167 µA and an isometric voxel size of 10 µm.

### Imaging software

**Figure 1:**
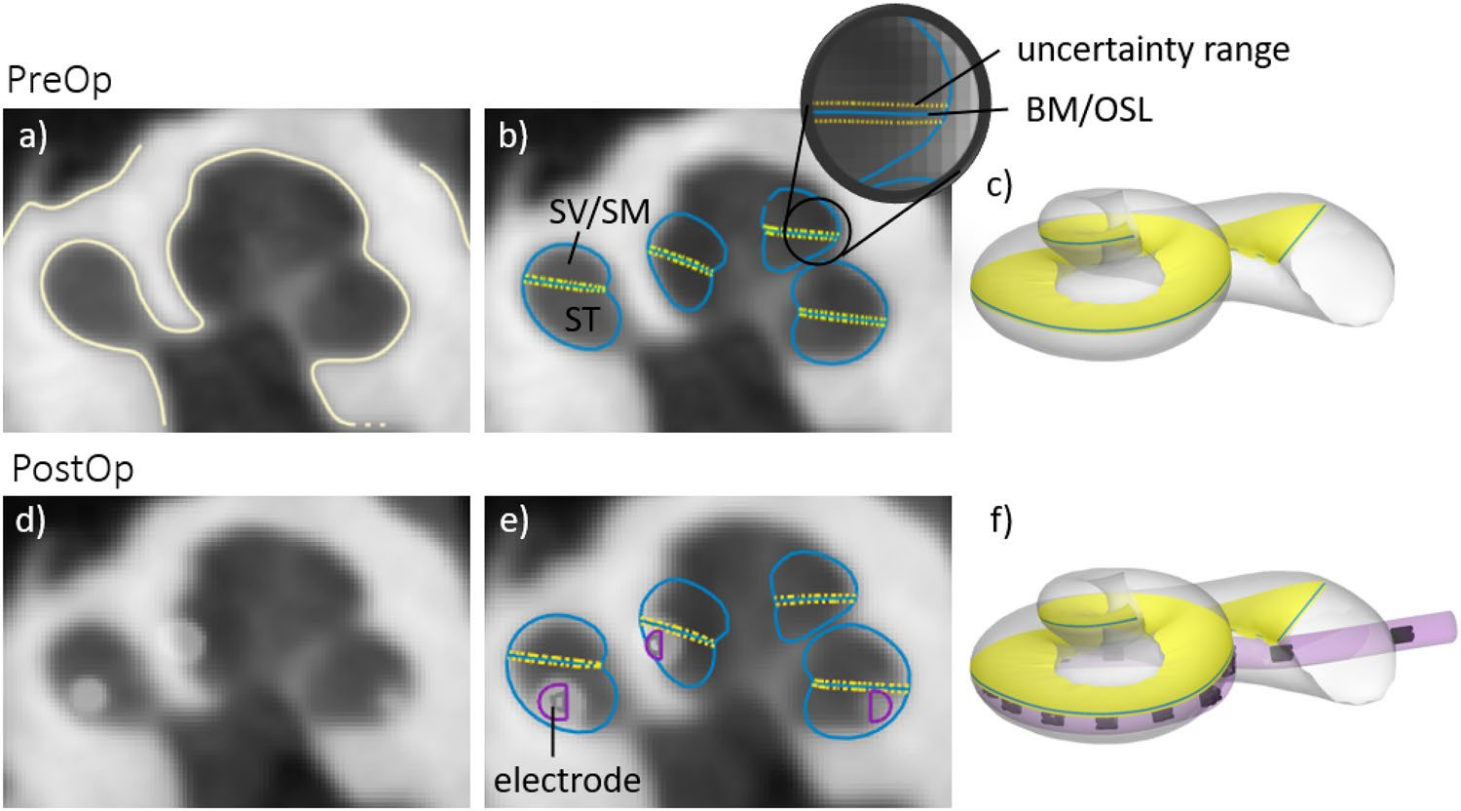
Image (**a**) shows the automatic segmentation of the cochlea. With a statistical shape model algorithm, a cochlear model (**c**) is fitted on the segmentation and superimposed on the CT scan (**b**). The model shows the scala tympani (ST), scala vestibuli and scala media (SV/SM) as well as the basilar membrane and osseous spiral lamina (BM/OSL) with the corresponding mean deviation as uncertainty range. Image (**d**) shows the postoperative CT scan registered on the preoperative CT scan. An algorithm detects the array contacts and generates a model of the electrode array (**e,f**).

A novel imaging software, developed by Advanced Bionics for research, was used to analyse preoperative and postoperative CBCT scans. The software is a MATLAB-based (MathWorks, Natick, MA) tool able to read and display sagittal, coronal and axial planes from multi-planar CT or CBCT scans. In addition, based on preoperative multi-planar CT or CBCT scans, the software is able to generate an individualised cochlear model with morphology and dimensions reflecting the anatomy of the specific cochlea. To do this, the software automatically detects the cochlea’s boundaries (Fig. 1a) within the CT scan and then uses a statistical shape model algorithm to generate the specific cochlear model (Fig. 1b,c). This model is generated using a dataset of 33 high-resolution cochlea models. These were created by manual reconstruction from high resolution micro-CT scans (10–16 µm voxel size). A number of 33 cochleae has been found sufficient to cover wide range of cochlear anatomies. The cochlear model consists of the scala tympani (ST) and the scala vestibuli/scala media (SV/SM) ducts, which are divided by the basilar membrane/osseous spiral lamina (BM/OSL), and are superimposed onto the CT image (Fig. 1b) and reconstructed in 3D (Fig. 1c). The BM/OSL is a critical anatomical structure for the evaluation of electrode array scalar location, so the algorithm has been designed with the aim of predicting the location of that structure as accurately as possible. As a result, an assessment of the error for the BM/OSL prediction has been conducted by comparing the model prediction by the software from a CT scan to the corresponding micro-CT scan. A set of ten of CT scans with corresponding micro-CTs were assessed by superimposing the generated model onto the corresponding micro-CT and measuring the distance between estimated BM/OSL to the actual position on the micro-CT. Measurements were performed on each set at seven locations distributed between 30 and 570 degrees and at each location measured laterally, centrally and modiolary, resulting in altogether 189 measurements. This assessment found a mean deviation of 0.06 mm. This upper and lower mean deviation is shown in yellow in Fig. 1b.

When loading the postoperative scans into the software, the software automatically registers the pre- and postoperative scans (Fig. 1d), detects the location of the SlimJ or MS electrode array contacts and generates a model of the electrode array (Fig. 1e,f). For each of the contacts, the software calculates the angular insertion depth and evaluates the electrode scalar location (see following section).

### Analysis of electrode scalar location

The automatic grading of electrode scalar location by the imaging software was compared to assessments made by two experienced otologists, familiar with evaluating pre- and postoperative CBCT scans performed in clinical routine. Only the CBCT was shown to the otologist without further guidance. An evaluation of the micro-CT scans was considered the ground truth. The scalar locations of the electrode contacts were evaluated perpendicular to the cochlea duct and at the center of each contact.

A simplified scale (Table 2) was introduced to rate the location of the electrode array based on clinical CT images. A rating of 0 means that the electrode sits at the observed location in the scala tympani. A rating of 1 indicates an interaction of the electrode with the basilar membrane or osseous spiral lamina (BM/OSL). This rating refers to potential trauma, as it can either mean a touching, elevation or rupture of the structures, which cannot be clearly determined from clinical scans. A rating of 2 means a translocation of the electrode into the scala media or vestibuli (SM/SV) and is defined as traumatic.

**Table 2:** The table shows the electrode scalar location grading (ESL).

Electrode scalar location (ESL) rating	Description
0	ST
1	Interaction with BM/OSL (potential trauma)
2	Translocation to SM/SV (trauma)

An ESL rating of 0 is associated with ‘Scala tympani (ST)’. A rating of 1 means an interaction of the electrode with the basilar membrane (BM) or osseus spiral lamina (OSL) occurred. A rating of 2 is used where a translocation of the electrode array into the scala media/vestibuli (SM/SV) occurred.

**Figure 2:**
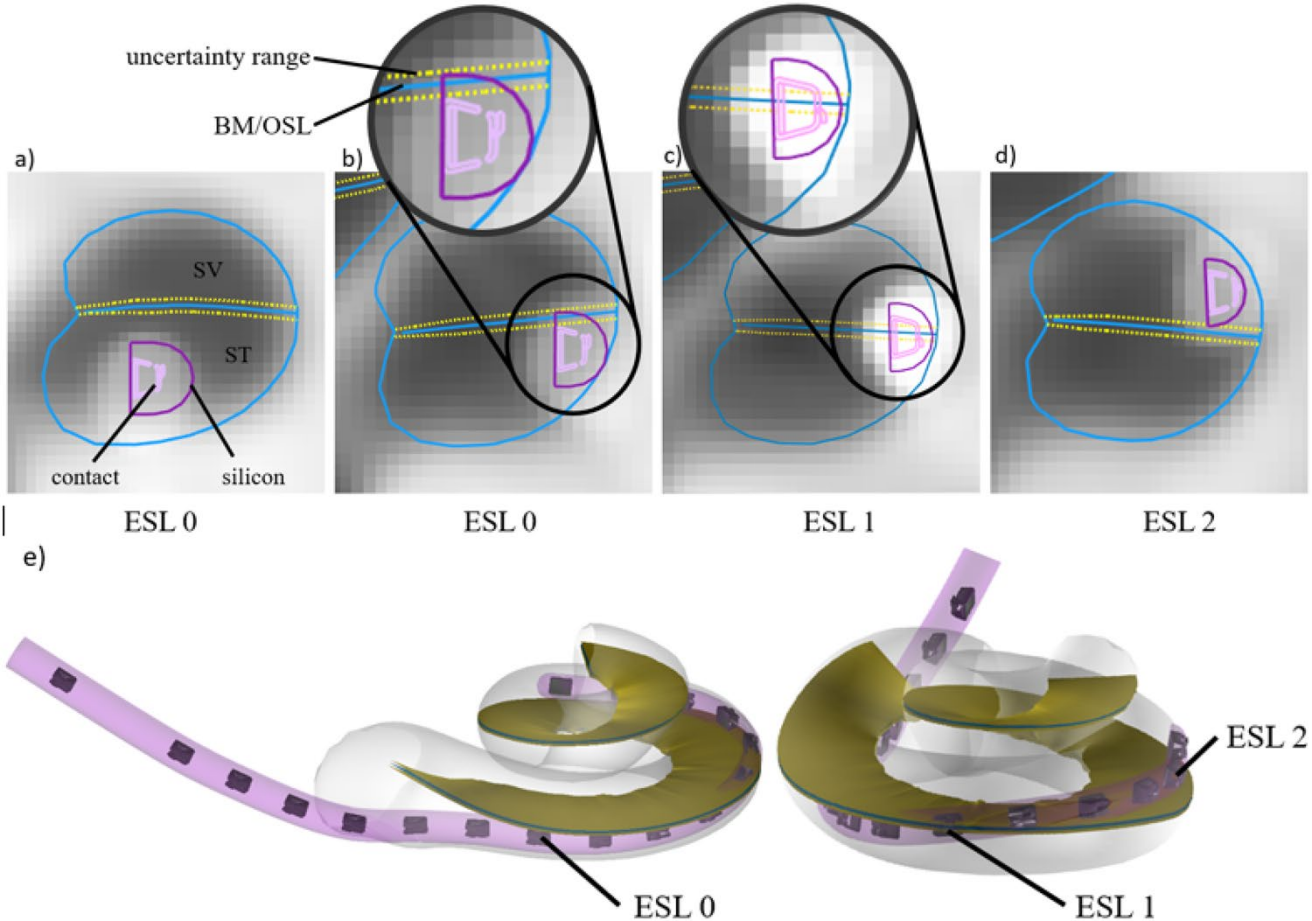
Images show the electrode scalar location (ESL) rating in 2D contour lines (**a–d**) and in 3D (**e**). Image (**a**) shows an ESL rating 0, image (**b**) is rated as 0 as the electrode is below the upper limit of the BM/OSL uncertainty range. Image (**c**) shows a rating of 1. Image (**d**) shows a rating of 2 as more than 50 percent of electrode is above the BM/OSL. The 3D view (**e**) shows the ESL as an example.

The software applies an ESL of 0 if the silicon electrode body sits below the upper limit of the BM/OSL uncertainty range (Fig. 2a,b). Once the silicon body is above the upper BM/OSL limit the rating is set to 1 (Fig. 2c). A rating of 2, is applied for a translocation of the electrode, defined by at least 50% of the electrode body siting above the BM/OSL estimation (Fig. 2d). Figure 2e shows an 3D example of the different ESL ratings.

### Statistics

Correlations between the different evaluation methods were determined using Cohen’s Kappa coefficient. The correlation test were performed with the Statistical Packages for the Social Sciences (SPSS, V25) for Windows (SPSS Inc., Chigaco, IL, USA).

The sensitivity and the specificity were calculated for the software’s results as well as results for both observers. The trauma rating based on the micro-CT images was considered as the ground truth. The sensitivity was defined by correct identification of an electrode contact’s scalar location rating of 1 and 2. As the ESL category 1 appears rarely, the trauma ratings of 1 and 2 were both combined. The specificity was defined as correctly identifying an ESL in the scala tympani (rating 0). Sensitivity and specificity equations are shown in Eqs. (1) and (2):

1 $$Sensitivity \left[\%\right]=\frac{number\, of\, correctly\, identified \,ESL\, ratings\, 1\, \&\, 2}{true \,number \,of\, ESL\, ratings\, 1\, \& \,2},$$

2 $$Specificity \left[\%\right]=\frac{number \,of \,correctly \,identified \,ESL \,rating\, 0}{true\, number\, of\, ESL\, ratings\, 0}.$$

### Ethical approval

The study fulfilled the Helsinki Declaration for Ethical use of human materials. The study was conducted according to Finnish legislation and institutional approval was granted by the Kuopio University Hospital (No. 5551883).

## Results

**Figure 3:**
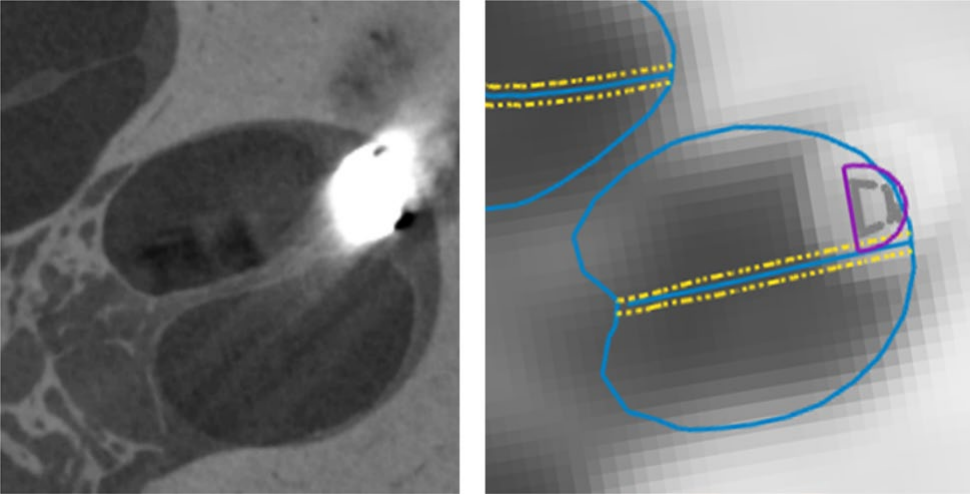
Shown is the TB2 at contact 4. On the left side is the micro-CT slice and on the right side the CBCT with the generated cochlea and electrode model superimposed. On both modalities a clear translocation (ESL = 2) can be observed.

Table 3 shows the results of the electrode contact scalar location ratings for all six temporal bones (TB1 to TB6). Ratings were color-coded in the table for ease of comparison (ESL 0: green, ESL 1: yellow, ESL 2: red). For each temporal bone, the software (SW) assessment and both observers’ (O1, O2) were compared to the ground truth of the micro-CT scans (µCT). TB1, TB3 and TB5 showed no translocation or interaction with the basilar membrane or osseous spiral lamina (BM/OSL). All ratings showed the same results as the µCT evaluation for these bones. TB2 (Fig. 3) and TB4 showed translocations of the electrode array with, in each case, one electrode contact indicating a BM/OSL interaction (rating 1) at the transition area. The software and both otologists correctly detected the translocated contacts, as well as the transition contact. TB6 did not show any trauma for the micro-CT rating. The software detected an interaction with the BM/OSL at contact 4 (C4). Observer 2 assessed C10 and C11 as having an interaction with the BM/OSL.

**Table 3:**
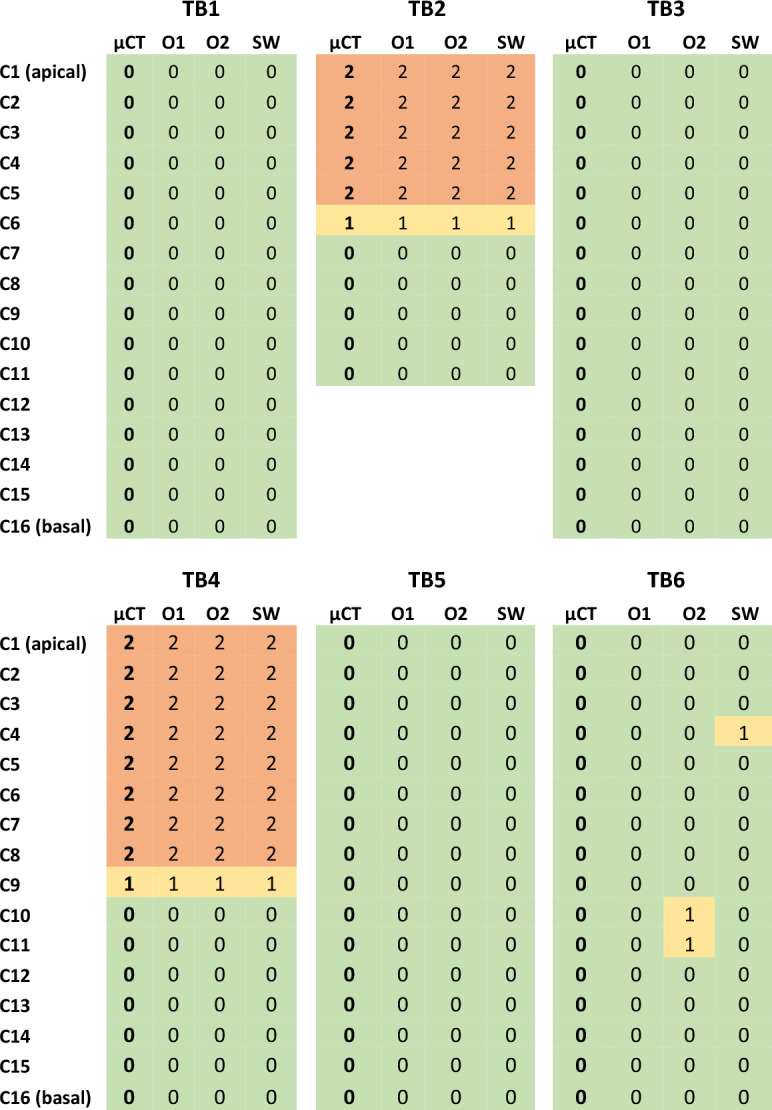
The table shows the electrode scalar location rating of the six temporal bones (TB).

Each contact (C1–C16) is rated. Ratings are shown for: the Micro-CT (µCT), observers 1 and 2 (O1, O2) and the software (SW). Virtually all ratings are the same across all four raters. Only for TB6 the software rates contact 4 (C4) as an interaction with the BM/OSL and observer 2 (O2) rates C10 and C11 as an interaction. Ratings were color-coded in the table for ease of comparison (ESL 0: green, ESL 1: yellow, ESL 2: red).

The software detected all ESL ratings of 1 and 2 correctly, resulting in a sensitivity of 100%. Since the software detected one 0 rating as an interaction, the specificity result was 98.7%. Observer 1 returned a 100% sensitivity as well as 100% specificity. Observer 2 returned a 100% sensitivity and a specificity of 97.4%. The combined results of both observers (O1 + O2) have 100% sensitivity and 98.7% specificity. The correlation between trauma grading methods was strong (kappa > 0.890).

**Figure 4:**
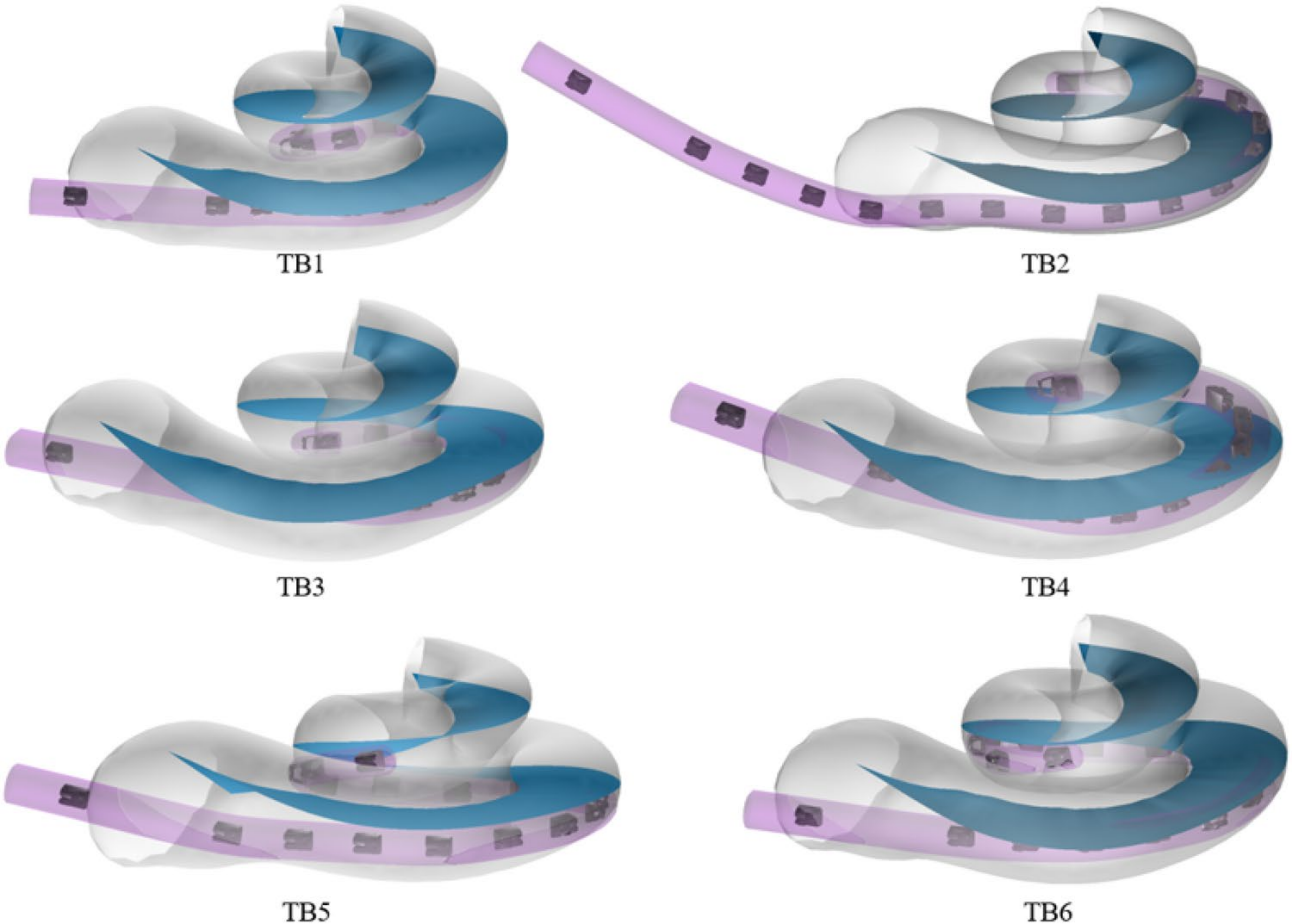
All six cochlear and electrode array models are shown. TB1, TB3, TB5 and TB6 do not show a translocation of the electrode array. For TB2, a partially inserted electrode, with a translocation into the scala vestibuli with a SlimJ array can be seen. TB4 shows a clear translocation with the MS electrode array.

All six generated cochlear and electrode array models are shown in Fig. 4. TB1 and TB3 show MS electrode arrays, implanted without translocation. For TB4 a translocation with the MS electrode array can be seen. TB5 and TB6 show SlimJ electrode arrays without translocation. TB2 shows a partially implanted electrode SlimJ array with a translocation. The 3D models generated for all six temporal bones are shown in Fig. 4.

## Discussion

In the present study, we introduced and evaluated a novel imaging software able to visualize and assess the success of CI insertion to ensure quality. The agreement between the imaging software with micro-CT and two observers was excellent with a sensitivity of 100% and specificity of 98.7%.

### Electrode scalar location scale

A widely used histological trauma grading scale was introduced by Eshraghi et al.^9^. This scale was developed to rate the intracochlear trauma caused by an implanted electrode array that was determined using histological images. Common clinical CT scans are often unable to reveal internal structures such as the osseous spiral lamina or the basilar membrane. This makes it necessary to develop an image-based grading scale, as no precise information about the internal structures can be derived. Several studies which assess electrode array scalar positions for clinical CTs, distinguish between the ratings ‘Electrode in Scala tympani’ (ST) and ‘Electrode in Scala vestibuli’ (SV)^10,15,19,23,26,30,31^. For an automatic assessment, a more precise scale was introduced by Torres et al.^21^ with the ratings ‘ST electrode’ with the criteria ≥ 50% of the electrode contact under the BM, ‘intermediate electrode’ with ≥ 10 to < 50% of the electrode contact under the BM and ‘SV electrode’ with < 10% of the electrode contact under the BM. Compared to Torres et al., the scale used in this study is stricter, as an electrode contact sitting 50% above the BM is already counted as a ‘translocation in SM/SV’ wherein the Torres et al. scale it would still be a ‘ST electrode’. As a result, it is more likely that trauma may go undetected with the Torres scale. Teymouri et al.^24^ uses a slightly stricter scale by defining a ST or SV position with the electrode contact being 75% below or above the BM/OSL respectively and as intermediate position if in between those values. This scale tolerates more potential trauma as the one, in the present study. Additionally, most past studies only reconstruct and consider the electrode contacts and the silicone body is neglected^23,24,26,21^, wherein in this study the silicone body is reconstructed with actual electrode array dimensions and also considered for potential trauma. Different rating scales, evaluation methods and electrode arrays make it difficult to directly compare the different assessments. Torres et al.^21^ reported on 15 implanted Advanced Bionics Mid-Scala electrode arrays an overall inter-rater agreement (Fleiss kappa = 0.68) between 3D-reconstruction assessment and histology. Teymouri et al.^24^ found an agreement between 3D-reconstruction assessment and histology of 94.9% evaluated on 158 electrode locations. For in vivo CT scans Sismono et al.^23^ and Andersen et al.^26^ reported 100% and 98.3% agreement between manual and automatic assessment, respectively. In those in vivo studies, however, no ground truth values, such as micro-CT or histology, were available. Furthermore, they only distinguished between ‘ST’ and ‘SV’ electrode location.

Considering the strict rating scale and the additionally modelled silicone body, this study indicates high accuracy in determining the scalar location and thus potential trauma to cochlear structures, given the high sensitivity and specificity.Sensitivity and specificity of the imaging software demonstrated similar accuracy to manual assessment by the experienced otologists. A high accuracy in predicting electrode array location and its potential impact on trauma is important to provide reliable and clinically relevant information.

### Evaluation time and observer experience

Another significant advantage of the software is the time required for the analysis. Users familiar with the software require between 7 and 12 min to complete the trauma grading analysis. Manual evaluation is highly dependent on the observer’s experience with image interpretation and the electrode location rating. In our test, manual analysis for one TB took about 20 min for the experienced observers.

Manual assessments require experience of the observer to achieve accurate results. To run an analysis with the novel software, only few steps need to be performed manually, so it can be used by less experienced users. The electrode scalar location analysis is made fully automatically. Other methods^24,27,21^ do require a lot of experience and manual corrections, which take too much time to be clinically applicable or results do depend on user input^21^ and are therefore, are only feasible for research purposes. The simple interface, as well as the fast analysis, makes the software applicable for clinical routine as well as large-scale studies.

Furthermore, the software generates a 3D model of both the cochlea and the electrode array. Although, this is not necessary in terms of trauma grading, the 3D model might provide valuable information, useful in understanding an electrode array’s location in relation to individual cochlear anatomies. It is a fast method that provides a quick overview of the insertion results and provides an evaluation of the electrode array’s location.

### Limitations

The accuracy of the imaging software intended for research use was evaluated using cadaveric temporal bones. This was necessary to obtain ground truth values from micro-CT images. In general, clinical CT images do have lower quality due to head shadows and possible movements during scanning as well as resolution varies for different clinical scanning protocols. Therefore, further investigation is needed in the clinical setting with regards to the software. Other limitations in this study are the small sample size and lack of histological samples.

## Conclusions

In this study, a novel imaging software was introduced to support the evaluation of electrode array scalar location based on clinical CBCT images. The software provided a fast and accurate assessment of electrode location within a human cochlea using a new radiologic scale for electrode location rating. Supported by the evidence found in this study, the software, in addition to research, may be also suitable for clinical use. The three-dimensional model allows a convenient and ‘at a glance’ evaluation of the electrode location and trauma. With a sensitivity of 100% and a specificity of 98.7%, the software provides a reliable electrode location rating that has been validated against a manual assessment by experienced otologists and micro-CT images, and may reduce the common inter-observer variation. The software could support large-scale evaluations for studies and also be of use in clinical routine.

## Data Availability

The datasets used and/or analyzed during the current study are available from the corresponding author on reasonable request.
